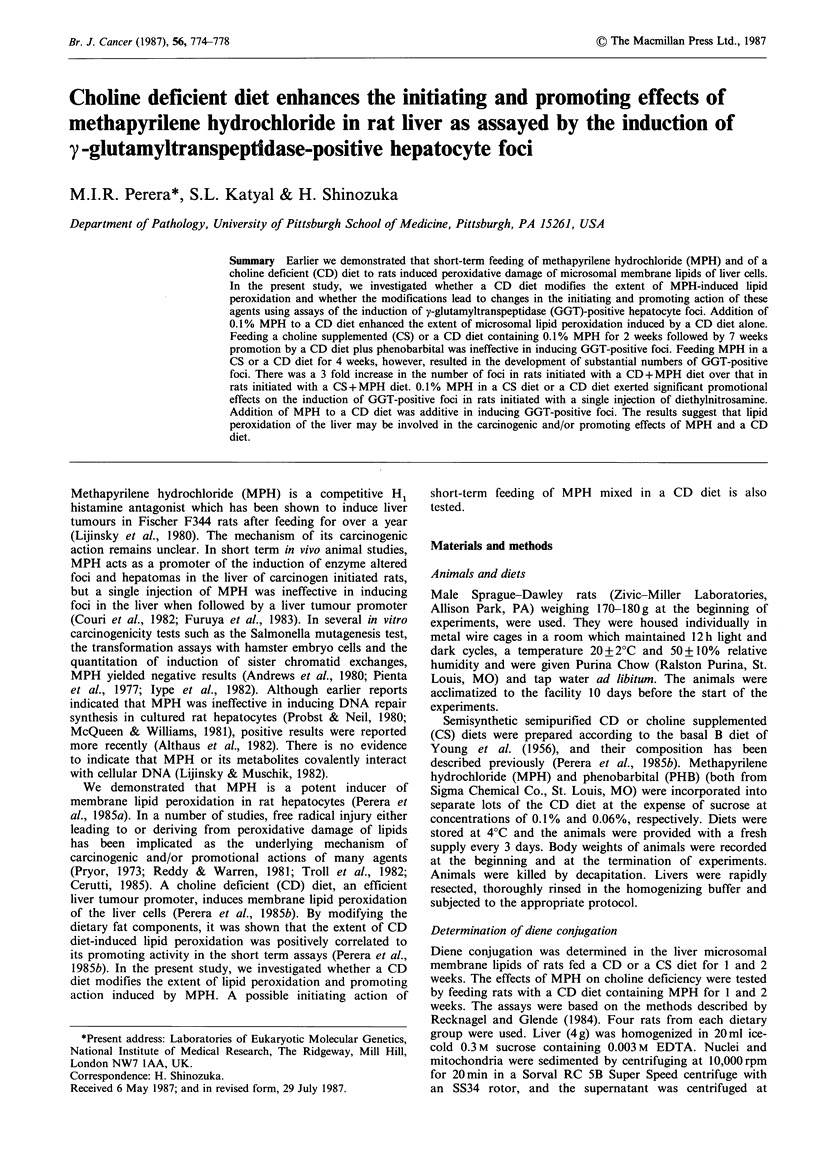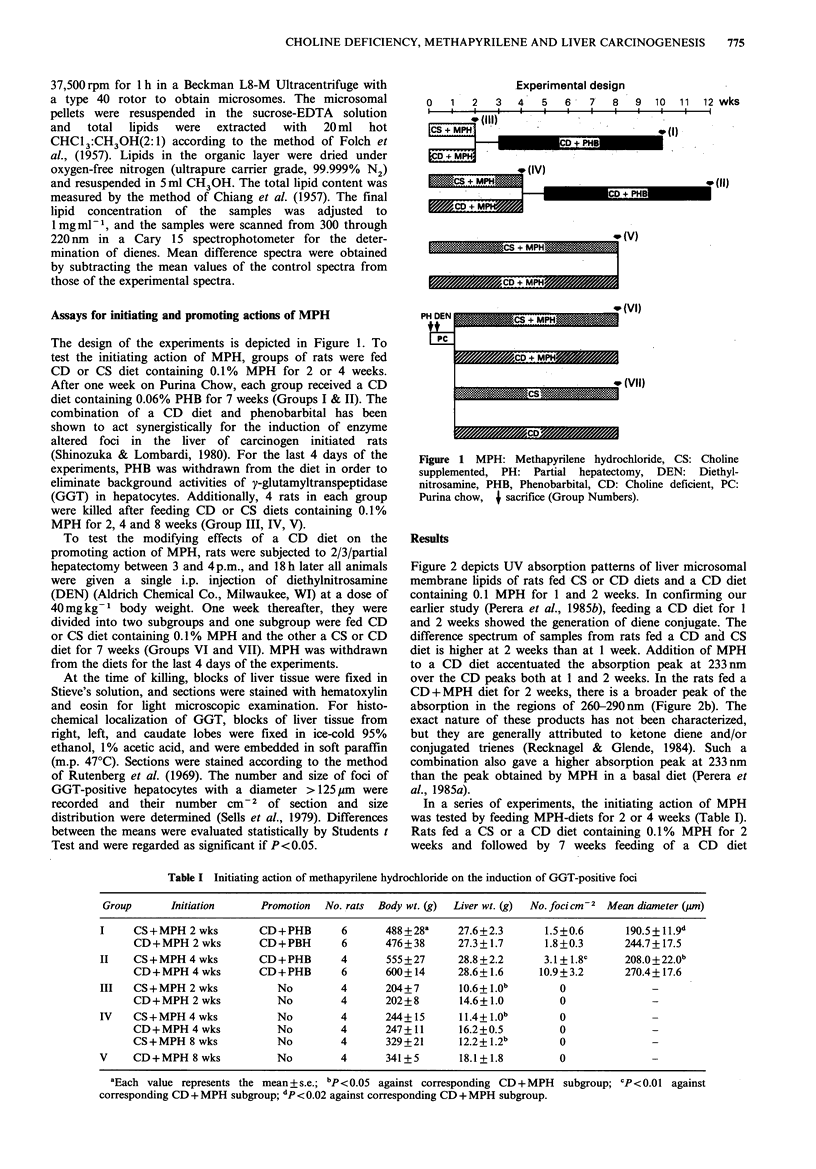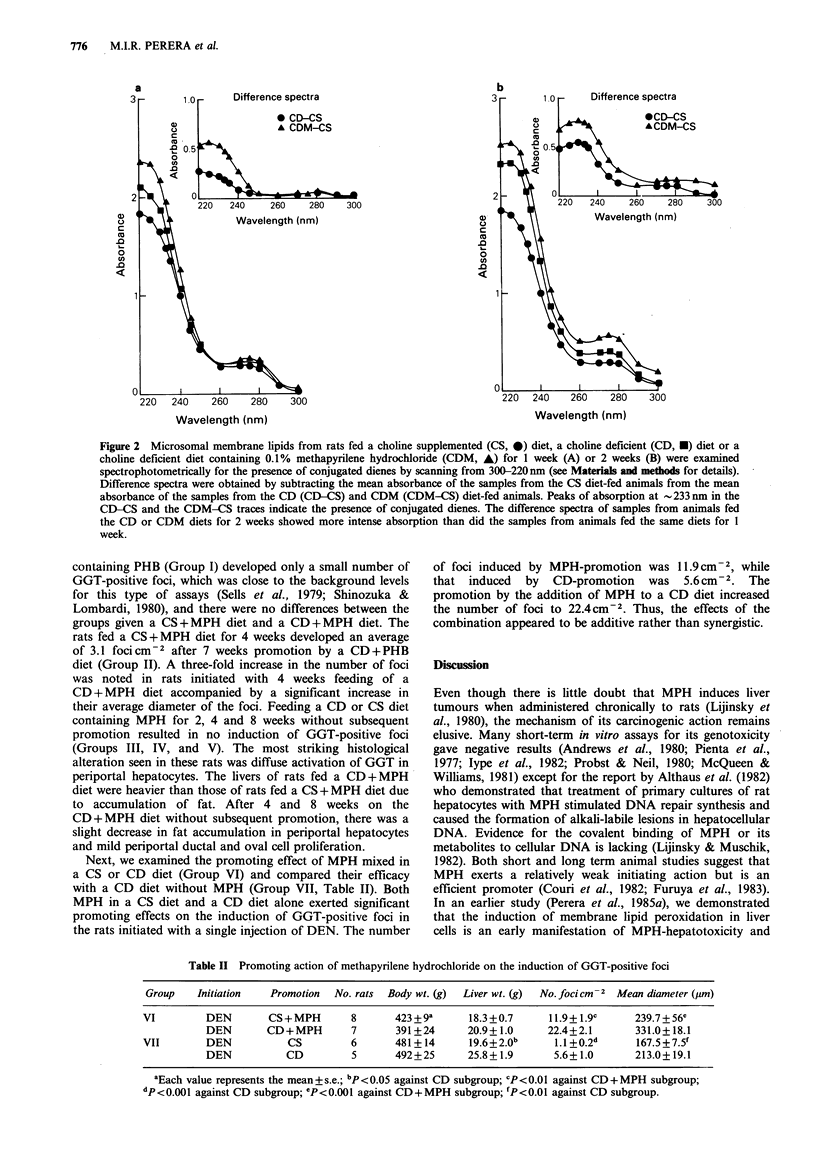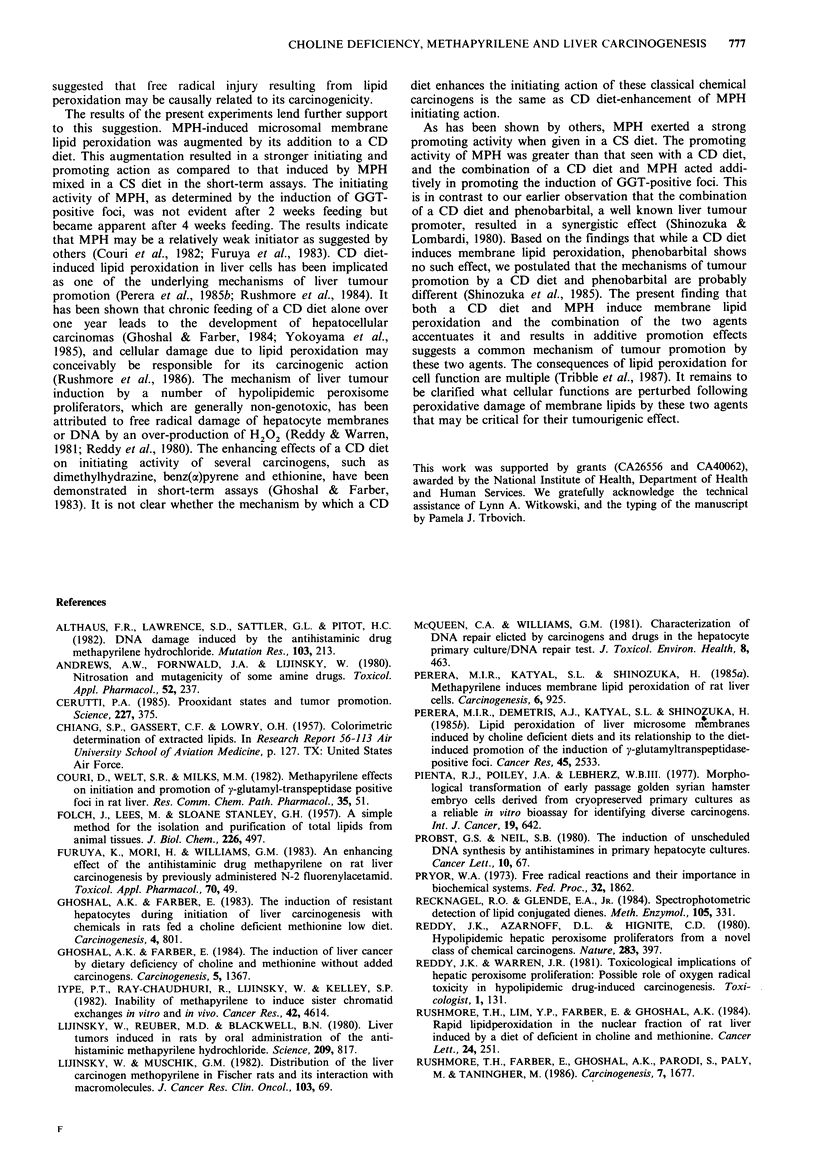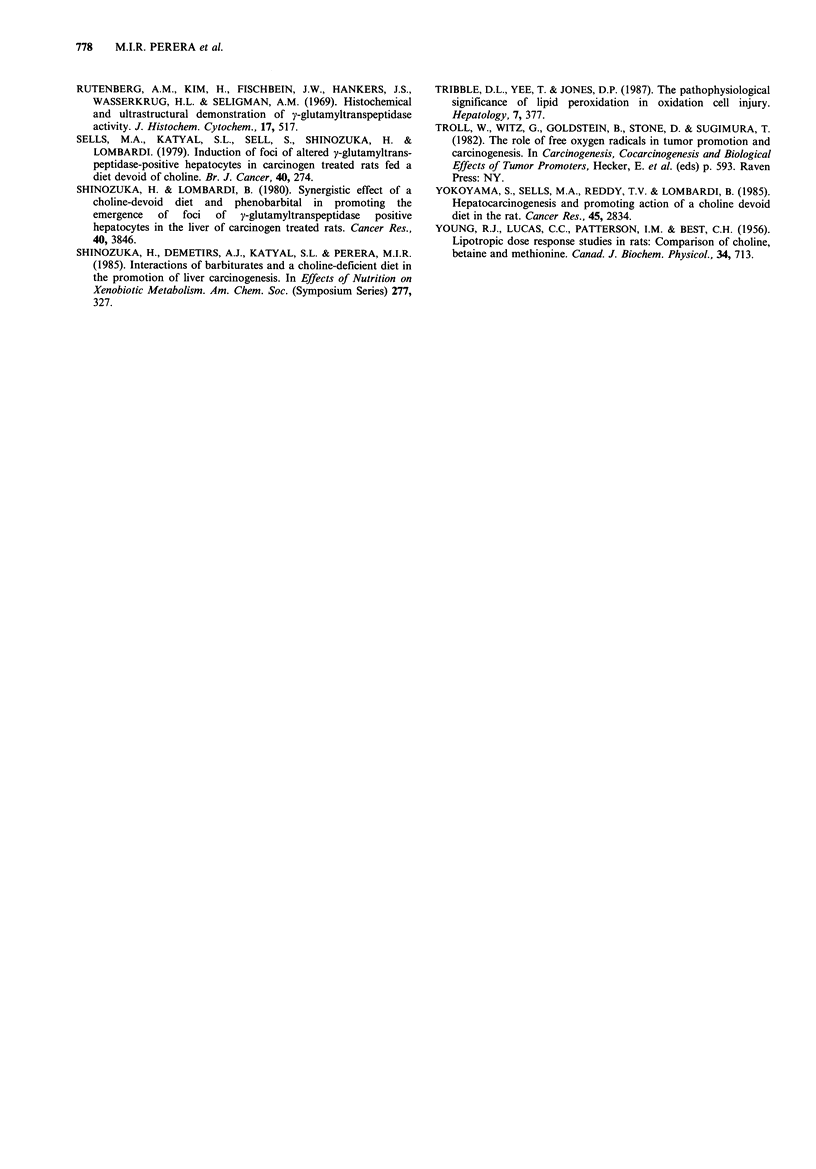# Choline deficient diet enhances the initiating and promoting effects of methapyrilene hydrochloride in rat liver as assayed by the induction of gamma-glutamyltranspeptidase-positive hepatocyte foci.

**DOI:** 10.1038/bjc.1987.286

**Published:** 1987-12

**Authors:** M. I. Perera, S. L. Katyal, H. Shinozuka

**Affiliations:** Department of Pathology, University of Pittsburgh School of Medicine, PA 15261.

## Abstract

Earlier we demonstrated that short-term feeding of methapyrilene hydrochloride (MPH) and of a choline deficient (CD) diet to rats induced peroxidative damage of microsomal membrane lipids of liver cells. In the present study, we investigated whether a CD diet modifies the extent of MPH-induced lipid peroxidation and whether the modifications lead to changes in the initiating and promoting action of these agents using assays of the induction of gamma-glutamyltranspeptidase (GGT)-positive hepatocyte foci. Addition of 0.1% MPH to a CD diet enhanced the extent of microsomal lipid peroxidation induced by a CD diet alone. Feeding a choline supplemented (CS) or a CD diet containing 0.1% MPH for 2 weeks followed by 7 weeks promotion by a CD diet plus phenobarbital was ineffective in inducing GGT-positive foci. Feeding MPH in a CS or a CD diet for 4 weeks, however, resulted in the development of substantial numbers of GGT-positive foci. There was a 3 fold increase in the number of foci in rats initiated with a CD + MPH diet over that in rats initiated with a CS + MPH diet. 0.1% MPH in a CS diet or a CD diet exerted significant promotional effects on the induction of GGT-positive foci in rats initiated with a single injection of diethylnitrosamine. Addition of MPH to a CD diet was additive in inducing GGT-positive foci. The results suggest that lipid peroxidation of the liver may be involved in the carcinogenic and/or promoting effects of MPH and a CD diet.


Br. J. Cancer (1987), 56, 774–778 © The Macmillan Press Ltd., 1987

### Choline deficient diet enhances the initiating and promoting effects of methapyrilene hydrochloride in rat liver as assayed by the induction of γ-glutamyltranspeptidase-positive hepatocyte foci

M.I.R. Perera*, S.L. Katyal & H. Shinozuka

*Department of Pathology, University of Pittsburgh School of Medicine, Pittsburgh, PA 15261, USA*

**Summary** Earlier we demonstrated that short-term feeding of methapyrilene hydrochloride (MPH) and of a choline deficient (CD) diet to rats induced peroxidative damage of microsomal membrane lipids of liver cells. In the present study, we investigated whether a CD diet modifies the extent of MPH-induced lipid peroxidation and whether the modifications lead to changes in the initiating and promoting action of these agents using assays of the induction of γ-glutamyltranspeptidase (GGT)-positive hepatocyte foci. Addition of 0.1% MPH to a CD diet enhanced the extent of microsomal lipid peroxidation induced by a CD diet alone. Feeding a choline supplemented (CS) or a CD diet containing 0.1% MPH for 2 weeks followed by 7 weeks promotion by a CD diet plus phenobarbital was ineffective in inducing GGT-positive foci. Feeding MPH in a CS or a CD diet for 4 weeks, however, resulted in the development of substantial numbers of GGT-positive foci. There was a 3 fold increase in the number of foci in rats initiated with a CD+MPH diet over that in rats initiated with a CS+MPH diet. 0.1% MPH in a CS diet or a CD diet exerted significant promotional effects on the induction of GGT-positive foci in rats initiated with a single injection of diethylnitrosamine. Addition of MPH to a CD diet was additive in inducing GGT-positive foci. The results suggest that lipid peroxidation of the liver may be involved in the carcinogenic and/or promoting effects of MPH and a CD diet.

Methapyrilene hydrochloride (MPH) is a competitive $H_1$ histamine antagonist which has been shown to induce liver tumours in Fischer F344 rats after feeding for over a year (Lijinsky *et al.*, 1980). The mechanism of its carcinogenic action remains unclear. In short term *in vivo* animal studies, MPH acts as a promoter of the induction of enzyme altered foci and hepatomas in the liver of carcinogen initiated rats, but a single injection of MPH was ineffective in inducing foci in the liver when followed by a liver tumour promoter (Couri *et al.*, 1982; Furuya *et al.*, 1983). In several *in vitro* carcinogenicity tests such as the Salmonella mutagenesis test, the transformation assays with hamster embryo cells and the quantitation of induction of sister chromatid exchanges, MPH yielded negative results (Andrews *et al.*, 1980; Pienta *et al.*, 1977; Iype *et al.*, 1982). Although earlier reports indicated that MPH was ineffective in inducing DNA repair synthesis in cultured rat hepatocytes (Probst & Neil, 1980; McQueen & Williams, 1981), positive results were reported more recently (Althaus *et al.*, 1982). There is no evidence to indicate that MPH or its metabolites covalently interact with cellular DNA (Lijinsky & Muschik, 1982).

We demonstrated that MPH is a potent inducer of membrane lipid peroxidation in rat hepatocytes (Perera *et al.*, 1985*a*). In a number of studies, free radical injury either leading to or deriving from peroxidative damage of lipids has been implicated as the underlying mechanism of carcinogenic and/or promotional actions of many agents (Pryor, 1973; Reddy & Warren, 1981; Troll *et al.*, 1982; Cerutti, 1985). A choline deficient (CD) diet, an efficient liver tumour promoter, induces membrane lipid peroxidation of the liver cells (Perera *et al.*, 1985*b*). By modifying the dietary fat components, it was shown that the extent of CD diet-induced lipid peroxidation was positively correlated to its promoting activity in the short term assays (Perera *et al.*, 1985*b*). In the present study, we investigated whether a CD diet modifies the extent of lipid peroxidation and promoting action induced by MPH. A possible initiating action of short-term feeding of MPH mixed in a CD diet is also tested.

*Present address: Laboratories of Eukaryotic Molecular Genetics, National Institute of Medical Research, The Ridgeway, Mill Hill, London NW7 1AA, UK.
Correspondence: H. Shinozuka.
Received 6 May 1987; and in revised form, 29 July 1987.

### Materials and methods

### *Animals and diets*

Male Sprague–Dawley rats (Zivic–Miller Laboratories, Allison Park, PA) weighing 170–180 g at the beginning of experiments, were used. They were housed individually in metal wire cages in a room which maintained 12 h light and dark cycles, a temperature 20±2°C and 50±10% relative humidity and were given Purina Chow (Ralston Purina, St. Louis, MO) and tap water *ad libitum*. The animals were acclimatized to the facility 10 days before the start of the experiments.

Semisynthetic semipurified CD or choline supplemented (CS) diets were prepared according to the basal B diet of Young *et al.* (1956), and their composition has been described previously (Perera *et al.*, 1985*b*). Methapyrilene hydrochloride (MPH) and phenobarbital (PHB) (both from Sigma Chemical Co., St. Louis, MO) were incorporated into separate lots of the CD diet at the expense of sucrose at concentrations of 0.1% and 0.06%, respectively. Diets were stored at 4°C and the animals were provided with a fresh supply every 3 days. Body weights of animals were recorded at the beginning and at the termination of experiments. Animals were killed by decapitation. Livers were rapidly resected, thoroughly rinsed in the homogenizing buffer and subjected to the appropriate protocol.

### *Determination of diene conjugation*

Diene conjugation was determined in the liver microsomal membrane lipids of rats fed a CD or a CS diet for 1 and 2 weeks. The effects of MPH on choline deficiency were tested by feeding rats with a CD diet containing MPH for 1 and 2 weeks. The assays were based on the methods described by Recknagel and Glende (1984). Four rats from each dietary group were used. Liver (4 g) was homogenized in 20 ml ice-cold 0.3 M sucrose containing 0.003 M EDTA. Nuclei and mitochondria were sedimented by centrifuging at 10,000 rpm for 20 min in a Sorval RC 5B Super Speed centrifuge with an SS34 rotor, and the supernatant was centrifuged at

37,500 rpm for 1 h in a Beckman L8-M Ultracentrifuge with a type 40 rotor to obtain microsomes. The microsomal pellets were resuspended in the sucrose-EDTA solution and total lipids were extracted with 20 ml hot $CHCl_3$:$CH_3OH$(2:1) according to the method of Folch *et al.*, (1957). Lipids in the organic layer were dried under oxygen-free nitrogen (ultrapure carrier grade, 99.999% $N_2$) and resuspended in 5 ml $CH_3OH$. The total lipid content was measured by the method of Chiang *et al.* (1957). The final lipid concentration of the samples was adjusted to 1 mg ml$^{-1}$, and the samples were scanned from 300 through 220 nm in a Cary 15 spectrophotometer for the determination of dienes. Mean difference spectra were obtained by subtracting the mean values of the control spectra from those of the experimental spectra.

**Assays for initiating and promoting actions of MPH**

The design of the experiments is depicted in Figure 1. To test the initiating action of MPH, groups of rats were fed CD or CS diet containing 0.1% MPH for 2 or 4 weeks. After one week on Purina Chow, each group received a CD diet containing 0.06% PHB for 7 weeks (Groups I & II). The combination of a CD diet and phenobarbital has been shown to act synergistically for the induction of enzyme altered foci in the liver of carcinogen initiated rats (Shinozuka & Lombardi, 1980). For the last 4 days of the experiments, PHB was withdrawn from the diet in order to eliminate background activities of $\gamma$-glutamyltranspeptidase (GGT) in hepatocytes. Additionally, 4 rats in each group were killed after feeding CD or CS diets containing 0.1% MPH for 2, 4 and 8 weeks (Group III, IV, V).

To test the modifying effects of a CD diet on the promoting action of MPH, rats were subjected to 2/3/partial hepatectomy between 3 and 4 p.m., and 18 h later all animals were given a single i.p. injection of diethylnitrosamine (DEN) (Aldrich Chemical Co., Milwaukee, WI) at a dose of 40 mg kg$^{-1}$ body weight. One week thereafter, they were divided into two subgroups and one subgroup were fed CD or CS diet containing 0.1% MPH and the other a CS or CD diet for 7 weeks (Groups VI and VII). MPH was withdrawn from the diets for the last 4 days of the experiments.

At the time of killing, blocks of liver tissue were fixed in Stieve's solution, and sections were stained with hematoxylin and eosin for light microscopic examination. For histochemical localization of GGT, blocks of liver tissue from right, left, and caudate lobes were fixed in ice-cold 95% ethanol, 1% acetic acid, and were embedded in soft paraffin (m.p. 47°C). Sections were stained according to the method of Rutenberg *et al.* (1969). The number and size of foci of GGT-positive hepatocytes with a diameter $>125\,\mu m$ were recorded and their number cm$^{-2}$ of section and size distribution were determined (Sells *et al.*, 1979). Differences between the means were evaluated statistically by Students *t* Test and were regarded as significant if $P<0.05$.

**Figure 1** MPH: Methapyrilene hydrochloride, CS: Choline supplemented, PH: Partial hepatectomy, DEN: Diethylnitrosamine, PHB, Phenobarbital, CD: Choline deficient, PC: Purina chow, ↓ sacrifice (Group Numbers).

**Results**

Figure 2 depicts UV absorption patterns of liver microsomal membrane lipids of rats fed CS or CD diets and a CD diet containing 0.1 MPH for 1 and 2 weeks. In confirming our earlier study (Perera *et al.*, 1985*b*), feeding a CD diet for 1 and 2 weeks showed the generation of diene conjugate. The difference spectrum of samples from rats fed a CD and CS diet is higher at 2 weeks than at 1 week. Addition of MPH to a CD diet accentuated the absorption peak at 233 nm over the CD peaks both at 1 and 2 weeks. In the rats fed a CD+MPH diet for 2 weeks, there is a broader peak of the absorption in the regions of 260–290 nm (Figure 2b). The exact nature of these products has not been characterized, but they are generally attributed to ketone diene and/or conjugated trienes (Recknagel & Glende, 1984). Such a combination also gave a higher absorption peak at 233 nm than the peak obtained by MPH in a basal diet (Perera *et al.*, 1985*a*).

In a series of experiments, the initiating action of MPH was tested by feeding MPH-diets for 2 or 4 weeks (Table I). Rats fed a CS or a CD diet containing 0.1% MPH for 2 weeks and followed by 7 weeks feeding of a CD diet

**Table I** Initiating action of methapyrilene hydrochloride on the induction of GGT-positive foci

| *Group* | *Initiation* | *Promotion* | *No. rats* | *Body wt. (g)* | *Liver wt. (g)* | *No. foci cm$^{-2}$* | *Mean diameter (μm)* |
|---|---|---|---|---|---|---|---|
| I | CS+MPH 2 wks | CD+PHB | 6 | 488±28[a] | 27.6±2.3 | 1.5±0.6 | 190.5±11.9[d] |
| | CD+MPH 2 wks | CD+PBH | 6 | 476±38 | 27.3±1.7 | 1.8±0.3 | 244.7±17.5 |
| II | CS+MPH 4 wks | CD+PHB | 4 | 555±27 | 28.8±2.2 | 3.1±1.8[c] | 208.0±22.0[b] |
| | CD+MPH 4 wks | CD+PHB | 6 | 600±14 | 28.6±1.6 | 10.9±3.2 | 270.4±17.6 |
| III | CS+MPH 2 wks | No | 4 | 204±7 | 10.6±1.0[b] | 0 | – |
| | CD+MPH 2 wks | No | 4 | 202±8 | 14.6±1.0 | 0 | – |
| IV | CS+MPH 4 wks | No | 4 | 244±15 | 11.4±1.0[b] | 0 | – |
| | CD+MPH 4 wks | No | 4 | 247±11 | 16.2±0.5 | 0 | – |
| | CS+MPH 8 wks | No | 4 | 329±21 | 12.2±1.2[b] | 0 | – |
| V | CD+MPH 8 wks | No | 4 | 341±5 | 18.1±1.8 | 0 | – |

[a]Each value represents the mean±s.e.; [b]$P<0.05$ against corresponding CD+MPH subgroup; [c]$P<0.01$ against corresponding CD+MPH subgroup; [d]$P<0.02$ against corresponding CD+MPH subgroup.

**Figure 2** Microsomal membrane lipids from rats fed a choline supplemented (CS, ●) diet, a choline deficient (CD, ■) diet or a choline deficient diet containing 0.1% methapyrilene hydrochloride (CDM, ▲) for 1 week (A) or 2 weeks (B) were examined spectrophotometrically for the presence of conjugated dienes by scanning from 300–220 nm (see **Materials and methods** for details). Difference spectra were obtained by subtracting the mean absorbance of the samples from the CS diet-fed animals from the mean absorbance of the samples from the CD (CD–CS) and CDM (CDM–CS) diet-fed animals. Peaks of absorption at ~233 nm in the CD–CS and the CDM–CS traces indicate the presence of conjugated dienes. The difference spectra of samples from animals fed the CD or CDM diets for 2 weeks showed more intense absorption than did the samples from animals fed the same diets for 1 week.

containing PHB (Group I) developed only a small number of GGT-positive foci, which was close to the background levels for this type of assays (Sells *et al.*, 1979; Shinozuka & Lombardi, 1980), and there were no differences between the groups given a CS+MPH diet and a CD+MPH diet. The rats fed a CS+MPH diet for 4 weeks developed an average of 3.1 foci $cm^{-2}$ after 7 weeks promotion by a CD+PHB diet (Group II). A three-fold increase in the number of foci was noted in rats initiated with 4 weeks feeding of a CD+MPH diet accompanied by a significant increase in their average diameter of the foci. Feeding a CD or CS diet containing MPH for 2, 4 and 8 weeks without subsequent promotion resulted in no induction of GGT-positive foci (Groups III, IV, and V). The most striking histological alteration seen in these rats was diffuse activation of GGT in periportal hepatocytes. The livers of rats fed a CD+MPH diet were heavier than those of rats fed a CS+MPH diet due to accumulation of fat. After 4 and 8 weeks on the CD+MPH diet without subsequent promotion, there was a slight decrease in fat accumulation in periportal hepatocytes and mild periportal ductal and oval cell proliferation.

Next, we examined the promoting effect of MPH mixed in a CS or CD diet (Group VI) and compared their efficacy with a CD diet without MPH (Group VII, Table II). Both MPH in a CS diet and a CD diet alone exerted significant promoting effects on the induction of GGT-positive foci in the rats initiated with a single injection of DEN. The number of foci induced by MPH-promotion was 11.9 $cm^{-2}$, while that induced by CD-promotion was 5.6 $cm^{-2}$. The promotion by the addition of MPH to a CD diet increased the number of foci to 22.4 $cm^{-2}$. Thus, the effects of the combination appeared to be additive rather than synergistic.

### Discussion

Even though there is little doubt that MPH induces liver tumours when administered chronically to rats (Lijinsky *et al.*, 1980), the mechanism of its carcinogenic action remains elusive. Many short-term *in vitro* assays for its genotoxicity gave negative results (Andrews *et al.*, 1980; Pienta *et al.*, 1977; Iype *et al.*, 1982; Probst & Neil, 1980; McQueen & Williams, 1981) except for the report by Althaus *et al.* (1982) who demonstrated that treatment of primary cultures of rat hepatocytes with MPH stimulated DNA repair synthesis and caused the formation of alkali-labile lesions in hepatocellular DNA. Evidence for the covalent binding of MPH or its metabolites to cellular DNA is lacking (Lijinsky & Muschik, 1982). Both short and long term animal studies suggest that MPH exerts a relatively weak initiating action but is an efficient promoter (Couri *et al.*, 1982; Furuya *et al.*, 1983). In an earlier study (Perera *et al.*, 1985*a*), we demonstrated that the induction of membrane lipid peroxidation in liver cells is an early manifestation of MPH-hepatotoxicity and

**Table II** Promoting action of methapyrilene hydrochloride on the induction of GGT-positive foci

| *Group* | *Initiation* | *Promotion* | *No. rats* | *Body wt.* (*g*) | *Liver wt.* (*g*) | *No. foci* $cm^{-2}$ | *Mean diameter* ($\mu m$) |
|---|---|---|---|---|---|---|---|
| VI | DEN | CS+MPH | 8 | 423±9[a] | 18.3±0.7 | 11.9±1.9[c] | 239.7±56[e] |
| | DEN | CD+MPH | 7 | 391±24 | 20.9±1.0 | 22.4±2.1 | 331.0±18.1 |
| VII | DEN | CS | 6 | 481±14 | 19.6±2.0[b] | 1.1±0.2[d] | 167.5±7.5[f] |
| | DEN | CD | 5 | 492±25 | 25.8±1.9 | 5.6±1.0 | 213.0±19.1 |

[a]Each value represents the mean±s.e.; [b]$P<0.05$ against CD subgroup; [c]$P<0.01$ against CD+MPH subgroup; [d]$P<0.001$ against CD subgroup; [e]$P<0.001$ against CD+MPH subgroup; [f]$P<0.01$ against CD subgroup.

suggested that free radical injury resulting from lipid peroxidation may be causally related to its carcinogenicity.

The results of the present experiments lend further support to this suggestion. MPH-induced microsomal membrane lipid peroxidation was augmented by its addition to a CD diet. This augmentation resulted in a stronger initiating and promoting action as compared to that induced by MPH mixed in a CS diet in the short-term assays. The initiating activity of MPH, as determined by the induction of GGT-positive foci, was not evident after 2 weeks feeding but became apparent after 4 weeks feeding. The results indicate that MPH may be a relatively weak initiator as suggested by others (Couri *et al.*, 1982; Furuya *et al.*, 1983). CD diet-induced lipid peroxidation in liver cells has been implicated as one of the underlying mechanisms of liver tumour promotion (Perera *et al.*, 1985*b*; Rushmore *et al.*, 1984). It has been shown that chronic feeding of a CD diet alone over one year leads to the development of hepatocellular carcinomas (Ghoshal & Farber, 1984; Yokoyama *et al.*, 1985), and cellular damage due to lipid peroxidation may conceivably be responsible for its carcinogenic action (Rushmore *et al.*, 1986). The mechanism of liver tumour induction by a number of hypolipidemic peroxisome proliferators, which are generally non-genotoxic, has been attributed to free radical damage of hepatocyte membranes or DNA by an over-production of $H_2O_2$ (Reddy & Warren, 1981; Reddy *et al.*, 1980). The enhancing effects of a CD diet on initiating activity of several carcinogens, such as dimethylhydrazine, benz(α)pyrene and ethionine, have been demonstrated in short-term assays (Ghoshal & Farber, 1983). It is not clear whether the mechanism by which a CD diet enhances the initiating action of these classical chemical carcinogens is the same as CD diet-enhancement of MPH initiating action.

As has been shown by others, MPH exerted a strong promoting activity when given in a CS diet. The promoting activity of MPH was greater than that seen with a CD diet, and the combination of a CD diet and MPH acted additively in promoting the induction of GGT-positive foci. This is in contrast to our earlier observation that the combination of a CD diet and phenobarbital, a well known liver tumour promoter, resulted in a synergistic effect (Shinozuka & Lombardi, 1980). Based on the findings that while a CD diet induces membrane lipid peroxidation, phenobarbital shows no such effect, we postulated that the mechanisms of tumour promotion by a CD diet and phenobarbital are probably different (Shinozuka *et al.*, 1985). The present finding that both a CD diet and MPH induce membrane lipid peroxidation and the combination of the two agents accentuates it and results in additive promotion effects suggests a common mechanism of tumour promotion by these two agents. The consequences of lipid peroxidation for cell function are multiple (Tribble *et al.*, 1987). It remains to be clarified what cellular functions are perturbed following peroxidative damage of membrane lipids by these two agents that may be critical for their tumourigenic effect.

This work was supported by grants (CA26556 and CA40062), awarded by the National Institute of Health, Department of Health and Human Services. We gratefully acknowledge the technical assistance of Lynn A. Witkowski, and the typing of the manuscript by Pamela J. Trbovich.

### References

ALTHAUS, F.R., LAWRENCE, S.D., SATTLER, G.L. & PITOT, H.C. (1982). DNA damage induced by the antihistaminic drug methapyrilene hydrochloride. *Mutation Res.*, **103**, 213.

ANDREWS, A.W., FORNWALD, J.A. & LIJINSKY, W. (1980). Nitrosation and mutagenicity of some amine drugs. *Toxicol. Appl. Pharmacol.*, **52**, 237.

CERUTTI, P.A. (1985). Prooxidant states and tumor promotion. *Science*, **227**, 375.

CHIANG, S.P., GASSERT, C.F. & LOWRY, O.H. (1957). Colorimetric determination of extracted lipids. In *Research Report 56-113 Air University School of Aviation Medicine*, p. 127. TX: United States Air Force.

COURI, D., WELT, S.R. & MILKS, M.M. (1982). Methapyrilene effects on initiation and promotion of γ-glutamyl-transpeptidase positive foci in rat liver. *Res. Comm. Chem. Path. Pharmacol.*, **35**, 51.

FOLCH, J., LEES, M. & SLOANE STANLEY, G.H. (1957). A simple method for the isolation and purification of total lipids from animal tissues. *J. Biol. Chem.*, **226**, 497.

FURUYA, K., MORI, H. & WILLIAMS, G.M. (1983). An enhancing effect of the antihistaminic drug methapyrilene on rat liver carcinogenesis by previously administered N-2 fluorenylacetamid. *Toxicol. Appl. Pharmacol.*, **70**, 49.

GHOSHAL, A.K. & FARBER, E. (1983). The induction of resistant hepatocytes during initiation of liver carcinogenesis with chemicals in rats fed a choline deficient methionine low diet. *Carcinogenesis*, **4**, 801.

GHOSHAL, A.K. & FARBER, E. (1984). The induction of liver cancer by dietary deficiency of choline and methionine without added carcinogens. *Carcinogenesis*, **5**, 1367.

IYPE, P.T., RAY-CHAUDHURI, R., LIJINSKY, W. & KELLEY, S.P. (1982). Inability of methapyrilene to induce sister chromatid exchanges *in vitro* and *in vivo*. *Cancer Res.*, **42**, 4614.

LIJINSKY, W., REUBER, M.D. & BLACKWELL, B.N. (1980). Liver tumors induced in rats by oral administration of the antihistaminic methapyrilene hydrochloride. *Science*, **209**, 817.

LIJINSKY, W. & MUSCHIK, G.M. (1982). Distribution of the liver carcinogen methopyrilene in Fischer rats and its interaction with macromolecules. *J. Cancer Res. Clin. Oncol.*, **103**, 69.

McQUEEN, C.A. & WILLIAMS, G.M. (1981). Characterization of DNA repair elicted by carcinogens and drugs in the hepatocyte primary culture/DNA repair test. *J. Toxicol. Environ. Health*, **8**, 463.

PERERA, M.I.R., KATYAL, S.L. & SHINOZUKA, H. (1985*a*). Methapyrilene induces membrane lipid peroxidation of rat liver cells. *Carcinogenesis*, **6**, 925.

PERERA, M.I.R., DEMETRIS, A.J., KATYAL, S.L. & SHINOZUKA, H. (1985*b*). Lipid peroxidation of liver microsome membranes induced by choline deficient diets and its relationship to the diet-induced promotion of the induction of γ-glutamyltranspeptidase-positive foci. *Cancer Res*, **45**, 2533.

PIENTA, R.J., POILEY, J.A. & LEBHERZ, W.B.III. (1977). Morphological transformation of early passage golden syrian hamster embryo cells derived from cryopreserved primary cultures as a reliable *in vitro* bioassay for identifying diverse carcinogens. *Int. J. Cancer*, **19**, 642.

PROBST, G.S. & NEIL, S.B. (1980). The induction of unscheduled DNA synthesis by antihistamines in primary hepatocyte cultures. *Cancer Lett.*, **10**, 67.

PRYOR, W.A. (1973). Free radical reactions and their importance in biochemical systems. *Fed. Proc.*, **32**, 1862.

RECKNAGEL, R.O. & GLENDE, E.A., JR. (1984). Spectrophotometric detection of lipid conjugated dienes. *Meth. Enzymol.*, **105**, 331.

REDDY, J.K., AZARNOFF, D.L. & HIGNITE, C.D. (1980). Hypolipidemic hepatic peroxisome proliferators from a novel class of chemical carcinogens. *Nature*, **283**, 397.

REDDY, J.K. & WARREN, J.R. (1981). Toxicological implications of hepatic peroxisome proliferation: Possible role of oxygen radical toxicity in hypolipidemic drug-induced carcinogenesis. *Toxicologist*, **1**, 131.

RUSHMORE, T.H., LIM, Y.P., FARBER, E. & GHOSHAL, A.K. (1984). Rapid lipidperoxidation in the nuclear fraction of rat liver induced by a diet of deficient in choline and methionine. *Cancer Lett.*, **24**, 251.

RUSHMORE, T.H., FARBER, E., GHOSHAL, A.K., PARODI, S., PALY, M. & TANINGHER, M. (1986). *Carcinogenesis*, **7**, 1677.

RUTENBERG, A.M., KIM, H., FISCHBEIN, J.W., HANKERS, J.S., WASSERKRUG, H.L. & SELIGMAN, A.M. (1969). Histochemical and ultrastructural demonstration of γ-glutamyltranspeptidase activity. *J. Histochem. Cytochem.*, **17**, 517.

SELLS, M.A., KATYAL, S.L., SELL, S., SHINOZUKA, H. & LOMBARDI. (1979). Induction of foci of altered γ-glutamyltranspeptidase-positive hepatocytes in carcinogen treated rats fed a diet devoid of choline. *Br. J. Cancer*, **40**, 274.

SHINOZUKA, H. & LOMBARDI, B. (1980). Synergistic effect of a choline-devoid diet and phenobarbital in promoting the emergence of foci of γ-glutamyltranspeptidase positive hepatocytes in the liver of carcinogen treated rats. *Cancer Res.*, **40**, 3846.

SHINOZUKA, H., DEMETIRS, A.J., KATYAL, S.L. & PERERA, M.I.R. (1985). Interactions of barbiturates and a choline-deficient diet in the promotion of liver carcinogenesis. In *Effects of Nutrition on Xenobiotic Metabolism. Am. Chem. Soc.* (Symposium Series) **277**, 327.

TRIBBLE, D.L., YEE, T. & JONES, D.P. (1987). The pathophysiological significance of lipid peroxidation in oxidation cell injury. *Hepatology*, **7**, 377.

TROLL, W., WITZ, G., GOLDSTEIN, B., STONE, D. & SUGIMURA, T. (1982). The role of free oxygen radicals in tumor promotion and carcinogenesis. In *Carcinogenesis, Cocarcinogenesis and Biological Effects of Tumor Promoters*, Hecker, E. *et al.* (eds) p. 593. Raven Press: NY.

YOKOYAMA, S., SELLS, M.A., REDDY, T.V. & LOMBARDI, B. (1985). Hepatocarcinogenesis and promoting action of a choline devoid diet in the rat. *Cancer Res.*, **45**, 2834.

YOUNG, R.J., LUCAS, C.C., PATTERSON, I.M. & BEST, C.H. (1956). Lipotropic dose response studies in rats: Comparison of choline, betaine and methionine. *Canad. J. Biochem. Physicol.*, **34**, 713.